# Topological FDR for neuroimaging

**DOI:** 10.1016/j.neuroimage.2009.10.090

**Published:** 2010-02-15

**Authors:** J. Chumbley, K. Worsley, G. Flandin, K. Friston

**Affiliations:** a Wellcome Trust Centre for Neuroimaging, 12 Queen Square, London; WC1N 3BG, UK; b Department of Statistics, University of Chicago, Chicago, IL, USA

## Abstract

In this technical note, we describe and validate a topological false discovery rate (FDR) procedure for statistical parametric mapping. This procedure is designed to deal with signal that is continuous and has, in principle, unbounded spatial support. We therefore infer on topological features of the signal, such as the existence of local maxima or peaks above some threshold. Using results from random field theory, we assign a *p*-value to each maximum in an *SPM* and identify an adaptive threshold that controls false discovery rate, using the Benjamini and Hochberg (BH) procedure (1995). This provides a natural complement to conventional family wise error (FWE) control on local maxima. We use simulations to contrast these procedures; both in terms of their relative number of discoveries and their spatial accuracy (via the distribution of the Euclidian distance between true and discovered activations). We also assessed two other procedures: cluster-wise and voxel-wise FDR procedures. Our results suggest that (a) FDR control of maxima or peaks is more sensitive than FWE control of peaks with minimal cost in terms of false-positives, (b) voxel-wise FDR is substantially less accurate than topological FWE or FDR control. Finally, we present an illustrative application using an fMRI study of visual attention.

## Introduction

Numerous authors have remarked that the fundamental effects of interest in fMRI are distributed and spatially continuous (e.g. Heller et al 2006, see also Pacifico et al 2004 and Benjamini and Heller 2007). A recent communication (Chumbley and Friston, 2009) proposed a solution based upon conditional *p*-values associated with the spatial extent of excursion sets above an arbitrary threshold (conditional on such a set existing). Here, we revisit the same issue and present an equivalent solution based on *p*-values associated with maxima or peaks in a Statistical Parametric Map (SPM). This allows us to assign FDR adjusted *p*-values (which we shall refer to as *q*-values) to both peaks and clusters within any SPM on image data.

Most instances of image analysis deal with signals that are continuous functions of some support; for example, anatomical space, time and frequency in EEG analyses or various combinations of space, time and frequency: In Chumbley and Friston (2009) we discuss why signal is continuous in the majority of neuroimaging applications and have reviewed the various image reconstruction procedures and biophysical mechanisms that generate continuous signals. If signal is a continuous function of the search space, strictly speaking, every discovery is a true discovery, because signal is everywhere. These conditions present a challenge to the conventional application of FDR to separate tests or voxels.

In Chumbley and Friston (2009) we described a solution based upon the topological features of the SPM. This involved controlling the false-discovery rate, not of voxels, but of connected components of the excursion set above some threshold (i.e., controlling the false-discovery rate of clusters). In this note, we pursue the same theme but control the false-discovery rate of maxima or peaks. Maxima or peaks are the most common topological features of activation profiles reported in the literature. Peaks are a useful characterisation of regionally specific responses because they have a well-defined location (usually reported in a standard anatomical space) and lend themselves to data-basing. Note that a simple^1^ suprathreshold region in an SPM could contain several peaks. Only at high thresholds, will the number of suprathreshold regions and peaks be equal. Usually, people display the SPM, at some threshold, as a maximum intensity projection or rendered image and then discuss peaks that survive a statistical criterion.

In what follows, we will consider signal to be a continuous function of a statistical search space and an effect or activation to be some topological feature of this function. In particular, we will consider an activation to be a peak of the signal and a discovery to be the corresponding peak in the statistical process or SPM. We thus convert a continuous process (signal) to a topological feature (activation) that does or does not exist. Finally, we may choose to define “true positive” and “false positive” topological decisions (with a latitude that is not possible in the definition of decisions in discrete tests). For example, one might reasonably label a discovered peak in an SPM “true positive” if it lies within one FWHM of a true peak in the underlying signal, and “false positive” otherwise. However, this categorisation of discoveries as true or false depends on an arbitrary threshold (here one FWHM). More generally then, we consider the full distribution on the distance between observed and true maxima under a procedure. This measure of the spatial accuracy is of interest *per se* and can be readily transformed back into a qualitative false-positive rate (by evaluating its cumulative density at any specified distance). To assess accuracy, one can compare these distributions of spatial error, over procedures.

This note comprises three sections. In the first, we present the theory behind the control for false-discovery rates of peaks. We then assess its performance in relation to conventional peak FWE in terms of two complementary measures: (a) the relative number of discoveries and (b) the spatial displacement of each discovered peak from the true underlying peak (defined as the minimum distance between true activations and discoveries). We report conditions under which the peak-BH procedure identifies more activations (as measured by a) at a negligible cost of identifying more false ones (as measured by b). This enables us to compare different procedures in quantitative terms, while side-stepping the different definitions of a discovery (e.g. of “activation”) under voxel-wise and topological approaches. In the final section, we apply the alternative procedures (FWE and FDR on peaks and FDR on clusters and voxels) to a standard fMRI dataset (the fMRI study of visual attention available at http://www.fil.ion.ucl.ac.uk/spm).

## Theory

In this section, we present the theory behind topological inference based on FDR for peaks. The usual approach to topological inference is as follows. First, derive the null distribution of the SPM, assuming no signal and Gaussian error fields. Then define a random set *A*_h,s_ of simple regions within the SPM, where each region exceeds *h* in height and *s* in spatial extent (*h*_*i*_ > *h*,*s_i_* > *s*)with *i* = 1, …, *M*_*h,s*_. Finally, derive the distribution of *M*_*h,s*_, under the assumptions described in Friston et al., 1996. This allows one to calibrate the thresholds (*h,s*) to control the expected number of false positives *E*(*M*_*h,s*_), or until the probability of getting any false positives *p*(*M*_*h,s*_ > 0) is sufficiently small; this is the family-wise error (FWE) rate. Having established the decision criterion (*h,s*) any observed region satisfying (*h*_*i*_ > *h,s_i_* > *s*) indicates the qualitative existence of one or more maxima in the underlying signal field (e.g., “an activation”).

An alternative approach, used in this work, views the random excursion set *A*_*h,s*_ as comprising two random subsets *A*_*h,s*_⊇ = {*A*_*h,s,f*_, *A*_*h,s,t*_} of false- and true-positive domains. The integer count of simple regions in *A*_*h,s*_ is now *M*_*h,s*_ = *M*_*h,s,f*_ + *M*_*h,s,t*_. We use a simple algorithm (Benjamini and Hochberg, 1995) to calibrate (*h,s*) so that *M*_*h,s,f*_/*M*_*h,s*_ < *α* on average. This approach does not seek to restrict the absolute number of false-positive regions (as above), but their fraction among all regions declared positive; this is the false discovery rate (FDR). This work (together with Chumbley and Friston, 2009) considers special cases, where either *h* or *s* are fixed in advance.

Briefly, we harvest the peaks above some user-specified threshold *u* and compute the *p*-values for each of these peaks. We then evaluate the corresponding *q*-values and threshold that controls the expected false-discovery rate (using Benjamini and Hochberg 1995). Note that we are controlling the false-discovery rate of peaks, not voxels. This critical distinction applies equally to the control of false-positive rates in conventional topological inference; namely, when we adjust the *p*-values for multiple comparisons using random field theory, we are controlling the false-positive rate of *peaks* or *maxima* within the SPM (not voxels): The expected number of false-positive voxels depends on the false-positive rate of peaks times the expected number of voxels subtending one peak. As the expected number of voxels per peak is always greater than one, the expected false-positive rate of voxels is always greater than the false-positive rate of peaks.

To compute the threshold for control of FDR, and associated *q*-values, we need the uncorrected *p*-value for each local peak in the SPM. We can compute the uncorrected *p*-value for peaks above some threshold using standard results from random field theory. This computation is based upon the fact that the *p*-value for a peak *Z* surpassing a threshold *u*, obtains by simply normalizing according to Bayes rule:

(1) $$p_{u}(z)=p(Z>z|Z>u)=\frac{p(Z>z)}{p(Z>u)}$$

Provided the regional excursions remain simple, the right hand side reports conditional *p*-values for *u* as follows.

(2) $$\begin{array}{l} p_{u}(z)\approx \frac{EC_{D}(z)}{EC_{D}(u)}\\ EC_{D}(u)>EC_{D}(z)\geq 0\Rightarrow 1\geq p_{u}(z)\geq 0 \end{array}$$

Here *EC*_*D*_ is the Euler characteristic density, the expected rate of emission of peaks per *resel* (a measure of effective volume after accounting for non-isotropic smoothness in a *D*-dimensional space). While there is an exact expression for the Euler characteristic at all *u* (Taylor and Worsley 2007), regions only remain simple for roughly *u* > 2.5.

In short, the uncorrected *p*-value is simply the ratio of the rates of getting peaks above *u* and *z*, which is available from standard results in random field theory (Worsley et al 1992, Friston et al 1994). For example, in the cases of *t* fields, with *v* degrees of freedom, the right hand side has the following form (see Worsley et al 1996),

(3) $$p_{u}(z)=\frac{{\left(1+\frac{z^{2}}{\nu }\right)}^{-1/2(\nu -1)}(\frac{\nu -1}{\nu }z^{2}-1)}{{\left(1+\frac{u^{2}}{\nu }\right)}^{-1/2(\nu -1)}(\frac{\nu -1}{\nu }u^{2}-1)}$$

These uncorrected *p*-values for maxima can then be submitted to an FDR-control algorithm (Benjamini and Hochberg 1995), which returns a threshold that controls the expected false-discovery rate. Note that a conventional FDR procedure would rank the uncorrected *p*-values associated with *all voxels*, treating them as a collection of discrete tests.

Generally, independence of the *p*-values and the number of tests is not needed in the BH procedure because the number of tests is fixed and the “distribution functions” are essentially deterministic. However, in our case, the number of *p*-values is itself a random variable because it corresponds to the number of maxima in an observed SPM. We therefore have to appeal to the Poisson clumping heuristic (Aldous, 1989) to motivate the assumption that the uncorrected *p*-values of the peaks are independent of their number. Intuitively, this means that the occurrence of a peak in one part of the SPM does not depend on the height of a peak in another part. Clearly, this rests on the smoothness of the SPM being smaller than its domain and, implicitly, a large number of peaks. Only under these circumstances is it sensible to control FDR. This means that the FDR procedures discussed above are only meaningfully applied when peaks are defined by a relatively low threshold on SPMs with a large number of *resels* (i.e., a high Lipschitz–Killing curvature; Taylor and Worsley 2007).

## Assessing FDR

We must take care when evaluating the performance of an FDR-control algorithm in the context of an alternative hypothesis that involves continuous signal with unbounded support. This is because under this condition, FDR on voxels is zero (because, strictly speaking, there is signal everywhere). We therefore use a performance measure based on topological features; namely, the smallest distance between the true activation (the peak of the signal) and the discoveries (peaks in the SPM). Notice that the “minimum distance” between a true activation and any peak in the SPM is well defined, even if the peak is not (e.g., a ridge of peaks) . However, this minimum distance measure is a pragmatic choice, which may confound attempts to put the numerical results presented below on a firm mathematical footing.

This minimum distance measure allows us to quantify the spatial specificity of the FDR-control procedure, without making strong assumptions about the nature of signal or its spatial support. In other words, by defining a true activation as a peak in the signal and a discovery as a supra-threshold peak in the SPM, we have a well-defined measure of the spatial relationship between the two that avoids defining a discovery in terms of voxels.

With this in mind, we define a vector-valued statistic *D* of the field under the alternative hypothesis (i.e., spatially smooth deterministic signal plus random field noise) such that the *i-*th element:$D_{i}=\min||X_{i}-\mu_{j}||:i=1,\ldots ,I$, where *I* is the (random) number of discoveries. $||X_{i}-\mu_{j}||$ is the Euclidian distance between the location of an *observed* local peak *X*_*i*_ and a *true* local peak in the underlying signal; the *μ*_*i*_ each belong to the set of true peak locations *Q*.

We can then consider the distribution of minimum distances, *p*_proc_(*d*_*i*_) corresponding to the random variables *D*_*i*_ associated with the discoveries from each procedure (proc). This distribution over the spatial displacements quantifies the spatial inaccuracy of a procedure. Using *Φ*_proc_(*d*_*i*_) to denote the cumulative density function of *p*_proc_(*d*_*i*_), 1-*Φ*_proc_(*d*_*i*_) reports the rate of displacements more extreme than *d*_*i*_.

In the next section, we compare topological FDR based upon peaks (peak-FDR) and clusters (cluster-FDR) and conventional voxel-wise FDR, based upon uncorrected voxel-wise *p*-values (voxel-FDR) against the benchmark of peak-FWE. We therefore contrast each procedure's displacement error with that of topological peak-FWE using:

(4) $$\Phi_{FWE}\left(d_{i}\right)-\Phi_{proc}\left(d_{i}\right)$$

This reports accuracy of a procedure (proc) relative to peak-FWE i.e. the rate of displacements more extreme than *d*_*i*_ attained using proc relative to that attained using standard peak-FWE control. To complement this measure, we also plotted *Φ*_FWE_(*d*_*i*_) against *Φ*_proc_(*d*_*i*_) (cf, a ROC curve). In these plots, a loss of spatial accuracy, relative to the FWE threshold means that for any distance, the number of discoveries *Φ*_proc_(*d*_*i*_) < *Φ*_FWE_(*d*_*i*_) will fall and the resulting curves will lie above the *Φ*_proc_ = *Φ*_FWE_ diagonal.

These two measures collectively summarize spatial accuracy. Note that spatial accuracy can always be increased at the expense of increased conservativeness by raising the height threshold *u*. This is because higher thresholds admit a fewer regions into the excursion set by chance, leaving a higher prevalence of regions with true underlying activation. Therefore, we also examined *r*_peak_ = dim(*D*_proc_) / dim(*D*_peakFWE_), the ratio of total peak-FDR and peak-FWE discoveries, irrespective of whether they are spatially accurate or not. As there are no convenient analytic results for *D*_*i*_, we proceed with simulations.

## Simulations and comparative evaluations

In this section, we present comparative evaluations of the different decision procedures using exactly the same simulated signal and error processes. We generated SPMs with 15 degrees of freedom by simulating 16 volumes of data and applying a voxel-wise *t* transformation. The data were a linear mixture of smooth signal and smooth noise. The noise was formed by convolving random number fields, sampled from a unit normal distribution, with a Gaussian kernel of four voxels in width. Signal was simulated by convolving (delta) stick functions with Gaussian kernels and adding them to the noise. We used a range of true activations *n*∈{2,4,8,16,32,64}, distributed uniformly at random over the search space. Each activation was generated by convolving a stick function at a randomly chosen voxel with a Gaussian kernel of variable width. The height of these functions was sampled from the chi-squared distribution with one degree of freedom. The width of the Gaussian kernel was sampled from a truncated Gamma distribution (truncated at four) of mean four and variance sixteen (shape and scale parameters of one and four respectively). The signal therefore comprised activations of positive height and varying width that was lower bounded by the smoothness of the noise.

We computed the adjusted *p*-values controlling family-wise error, the uncorrected voxel, peak and cluster *p*-values and their BH thresholds. The last two *p*-values require the pre-specification of a height threshold *u*, which we varied between 2.5 and 5.5. We then computed *D*_*i*_, the distance to the nearest true activation, for each activation discovered by the peak-FWE, peak-FDR, cluster-FDR, voxel-FDR procedures; all at 0.05 threshold. To ensure the distributions of distances were comparable across the four thresholding techniques, we only considered distances between true activations and peaks in the SPMs. This means that we ignored voxels in the voxel-FDR analysis that were not maxima and included all peaks in significant clusters in the cluster-FDR. Note that generally, one would elect to control either FDR on clusters (i.e., regions) or peaks. Given that clusters and peaks are distinct topological features, the ensuing inferences are categorically different. However, our assessment of spatial accuracy can be applied to both clusters and peaks because each cluster must contain one or more peaks.

We simulated 2000 SPMs and accumulated the distribution of minimum distances, *p*_proc_(*d*_*i*_) corresponding to the random variables *D*_*i*_ associated with the discoveries from each procedure (proc).

## Results

**Fig. 1:**
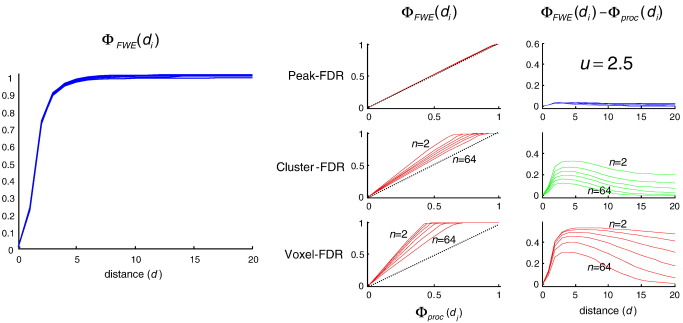
This figure shows the rate at which, in comparison to peak-FWE, peaks identified by various FDR procedures lie beyond a distance *d* in mm of the true signal peak*.* These results suggest uniformly higher spatial errors for all FDR procedures. For topological FDR, the relative displacement errors are small: Left: the cumulative density function (*cdf*) of displacement errors from conventional peak-FWE under *n* = 2, 4, 8,16, 32, 64 true activations. Right: the first column plots the *cdf* from the FWE procedure against the *cdf* of the FDR procedures (in the case of peak- and cluster-FDR, the feature-inducing threshold *u* = 2.5). A procedure has less spatial accuracy than peak-FWE when these functions lie above the dotted lines. The second column plots the difference in cumulative densities as a function of distance. Here, inaccuracy means a difference that is greater than zero. The three rows compare peak-FWE with peak-FDR, cluster-FDR and voxel-FDR.

**Fig. 2:**
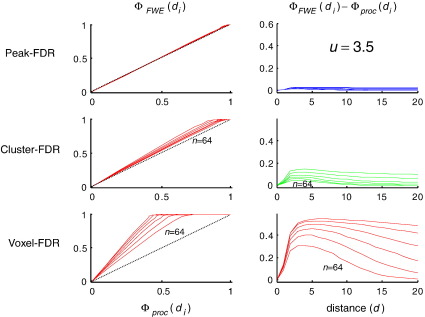
Same as the right panels in Fig. 1 but using a threshold of *u* = 3.5.

**Fig. 3:**
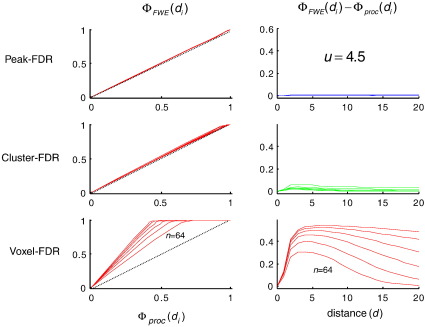
Same as Fig. 2 but using a threshold of *u* = 4.5.

**Fig. 4:**
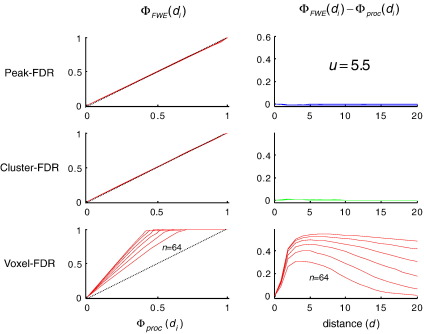
Same as Fig. 2 but using a threshold of *u* = 5.5.

Fig. 1 (left) shows *Φ*_FWE_(*d*_*i*_), the cumulative distribution function of minimum distance from observed to true maxima as disclosed by peak-FWE control. It can be seen that 95% of discovered activations fall within a FWHM of a true maxima in the underlying signal, irrespective of the number of maxima in the underlying simulation (plots for *n* = 2, 4, 8, 16, 32, 64 are superimposed). Rows in the right hand panel (Fig. 1) give two complementary measures that contrast spatial accuracy of (a) peak-FDR, (b) cluster-FDR and (c) voxel-FDR with respect to the peak-FWE reference. The former two require a feature-defining threshold, here 2.5. Irrespective of the number of activations in the underlying signal they show that peak-FDR is less accurate than cluster-FDR, which is in turn less accurate than peak-FDR. The latter is practically indistinguishable in its spatial accuracy from peak-FWE. As the threshold is increased to 3.5, 4.5, 5.5 (Figs. 2, 3, 4) the spatial accuracy of cluster-FDR increases. The spatial accuracy of peak-FDR with respect to peak-FWE is at ceiling with respect to peak-FWE throughout.

**Fig. 5:**
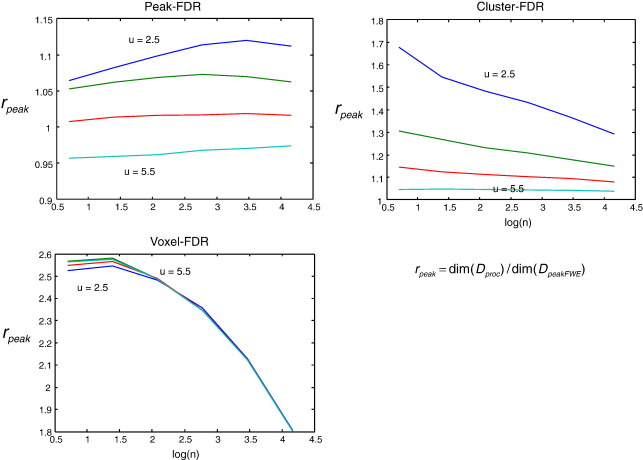
These plots describe the relative number of discoveries with three procedures; peak-FDR (top left), cluster-FDR (top right) and voxel-FDR (bottom left), compared to conventional peak-FWE. Each plot describes the dependence of this relative-sensitivity measure on the number of underlying activations (*n* = 2, 4, 8, 16, 32, 64) under a range of feature-inducing thresholds (*u* = 2.5, 3.5, 4.5, 5.5 ). For any threshold *u* and any number of activations *n*, voxel-FDR is more sensitive than cluster-FDR, which in turn is more sensitive than peak-FDR. Providing the feature-inducing threshold *u* is not too high (here approximately *u* < 5) peak-FDR is more sensitive than peak-FWE.

To characterise the sensitivity of the FDR procedures relative to the peak-FWE procedure, we then examined *r*_peak_, the ratio of total discoveries. Fig. 5 shows that for any threshold *u* and any number of activations *n*, voxel-FDR is more sensitive than extent-FDR, which in turn is more sensitive than peak-FDR. Peak-FDR tends to become more conservative with higher thresholds or fewer true activations. Providing the feature-inducing threshold *u* is not too high (here approximately *u* < 5) peak-FDR is more sensitive than peak-FWE (top left). The imperative to use a low threshold must, of course, be balanced against the assumptions of random field theory, which require a reasonably high threshold.

In summary, these results validate peak-FDR and illustrate the conditions under which it is more sensitive than conventional FWE control. This increased sensitivity incurs a negligible cost in terms of accuracy or false positives (see Fig. 1). Strictly speaking, the true voxel-wise FDR must be zero if the signal is unbounded. However, there is nothing to prevent us considering the voxel-wise BH procedure as a candidate for furnishing statistical control over topological inferences (here the number of peaks in the excursion set). Evaluated from this perspective we see that conventional voxel-FDR discovers more local peaks than conventional FWE control (Fig. 4), but only at the expense of spatial accuracy (or failure to control discoveries that are false under a spatial criterion).

## An illustrative application

In this section, we illustrate the application of the four procedures above to an empirical fMRI data set. We use a standard data set, which is available from http://www.fil.ion.ucl.ac.uk/spm, so that readers can reproduce the analyses below. These data have been used previously to illustrate various developments in data analysis.

Subjects were studied with fMRI under identical stimulus conditions (visual motion subtended by radially moving dots) under different attentional tasks (detection of velocity changes). The data were acquired from normal subjects at 2-Tesla using a Magnetom VISION (Siemens, Erlangen) whole-body MRI system, equipped with a head volume coil. Contiguous multi-slice T2⁎-weighted fMRI images were obtained with a gradient echo-planar sequence (TE = 40 ms, TR = 3.22 s, matrix size = 64 × 64 × 32, voxel size 3 × 3 × 3 mm). The subjects had four consecutive hundred-scan sessions comprising a series of ten-scan blocks under five different conditions D F A F N F A F N S. The first condition (D) was a dummy condition to allow for magnetic saturation effects. F (Fixation) corresponds to a low-level baseline where the subjects viewed a fixation point at the centre of a screen. In condition A (Attention) subjects viewed 250 dots moving radially from the centre at 4.7 degrees per second and were asked to detect changes in radial velocity. In condition N (No attention) the subjects were asked simply to view the moving dots. In condition S (Stationary) subjects viewed stationary dots. The order of A and N was swapped for the last two sessions. In all conditions, subjects fixated the centre of the screen. In a pre-scanning session the subjects were given five trials with five speed changes (reducing to 1%). During scanning there were no speed changes. No overt response was required in any condition. Data from the first subject are used here. We smoothed the data with Gaussian FWHM of 4, which is typical for single-subject fMRI.

**Fig. 6:**
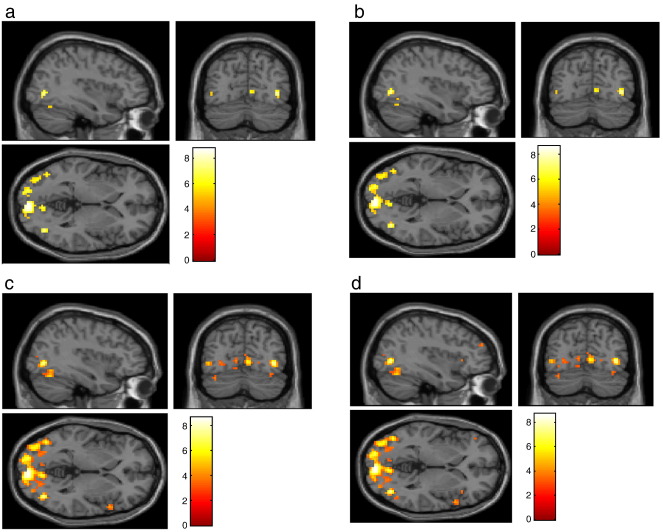
SPMs thresholded according to (a) peak-FWE (b) peak-FDR (c) cluster-FDR (d) voxel-FDR.

Fig. 5 shows the results of conventional SPM analyses, using a linear convolution model formed by convolving box-car stimulus functions with a canonical hemodynamic response function and its temporal derivative. The stimulus functions encoded the presence of photic stimulation, visual motion and attention. The *SPM*s in Fig. 6 test for an effect of motion and in trans-axial slices though V5 or human MT. The four *SPM*s are thresholded at 0.05 using peak-FWE, peak-FDR, cluster-FDR and voxel-FDR. The feature defining threshold was *u* = 3.

These SPMs recapitulate the general features seen in the simulations. The number of peaks in the whole brain surviving each of these thresholds increases monotonically from 27 (peak-FWE) to 96 peaks (voxel-FDR): peak-FWE < peak-FDR < cluster-FDR < voxel-FDR.

## Discussion

In this note, we have presented a topological FDR procedure that can be applied to peaks in an SPM. This is homologous to equivalent random field theory adjustments that assign corrected *p*-values to maxima and thereby control the false-positive rate of peaks. This procedure finds more peaks than FWE with a negligible cost in terms of spatial accuracy. In comparison with conventional voxel-wise FDR-control procedures, the peak-wise FDR control has fewer false positives and can be applied when signal or treatment effects are distributed in a continuous fashion over tests of voxels.

To quantify the performance of false-discovery rate schemes one needs to define, operationally, what one means by a true or false discovery. This is, in fact, a difficult problem in the context of continuous signals because there is no necessary one-to-one relation between the topological features of signal and the associated SPM. This is true irrespective of the multiple comparisons procedure. In other words, even using conventional control over family-wise error, one can never make an inference about where a signal came from. However, anecdotally, people usually associate the location of a peak in the SPM with the source of the underlying signal. We have tried to capture this anecdotal association by looking at the distribution of the distances between true activations and discoveries, under a number of different thresholding procedures. This avoids labelling voxels as true or false, while using a metric that relates the discoveries to true underlying activations. One might have defined a discovery as true or false by simply thresholding the known signal and requiring true discoveries to lie over thresholded signal. However, this implicitly defines an activation as signal above some specified threshold, which is unknown. Furthermore, we could arbitrarily adjust the true and false discovery rates by changing the threshold used to define activation. For example, a very small threshold would mean that all discoveries were true discoveries and the FDR would be zero. Conversely, a very high threshold would mean that all discoveries were false and the FDR would be 100%. The advantage of using topological features like maxima is that they do not require an arbitrary threshold on signal for their definition. Having said this, our uncorrected *p*-values do require a threshold to define an excursion set for subsequent topological FDR control. However, this threshold is applied *post hoc* to the statistical process, not to the signal.

We have emphasised the implications of signal with unbounded support because this lies at the heart of topological inference and distinguishes statistical parametric mapping from conventional approaches to large families of statistical tests. In a conventional setting, each test (i.e., voxel) can be declared significant. This is not the case in topological inference because only topological features are tested. This is called for when treatment effects (e.g., activations) are distributed in a continuous way over tests (e.g., voxels). This is what topological inference (SPM) was developed for. However, signals do not always have unbounded support; and even if they did mathematically, they may not in practice (e.g., after smoothing, the height of a signal will be trivially small a few FWHM from its centre. Similarly, finite-support smoothing preserves compactness; i.e. some voxels may have no signal after smoothing). Does this mean topological FDR is contraindicated when smoothness is small? No, a small smoothness means the number of peaks increases and topological FDR will approximate the results of conventional FDR control on voxels. However, the converse is not true; if the signal is distributed, conventional FDR (as currently applied in neuroimaging) is compromised. This is because the FDR of voxels is generally smaller than the FDR of regional responses, for any given threshold.

The concept of distributed signal is not introduced to undermine the appropriate use of voxel-wise methods in certain cases: “All models are wrong, but some are useful” (Box and Draper, 1987). However, if continuous regional effects are of interest, then one requires statistical error-control of regional inferences. The regional error-control performance of voxel-wise methods can be verified *post-hoc* on a case-by-case basis (according to some definition of regional activation). For example, voxel-wise Bonferroni control incidentally guarantees error-control on regional peaks as we have defined them (e.g. Nichols and Hayasaka, 2003). Conversely, voxel-wise methods may fail to control regional error rates in other contexts (Chumbley and Friston, in press ).

In conclusion, we have supplemented our previous report of FDR control based upon the spatial extent of clusters to include a procedure that assigns FDR *q*-values to maxima within SPMs. We have quantified the performance of this procedure in terms of the spatial specificity of the ensuing discoveries and, providing the excursion threshold is sufficiently low, found it to perform largely as expected. Our conclusions, however, depend upon the particular model for signal used in our simulations. This dependency highlights the difficulty of assessing FDR procedures in general and, in particular, the sorts of signals entailed by neuroimaging.

## Software note

The topological FDR procedures based upon spatial extent and peak values will be implemented in SPM8 and SurfStat, which is available as academic freeware from http://wwww.fil.ion.ucl.ac.uk/SPM and http://www.math.mcgill.ca/keith/surfstat.

## Footnotes

1 Connected excursion sets with no holes, handles, etc.
